# Gancao Nourishing-*Yin* decoction combined with methotrexate in treatment of aging CIA mice: a study based on DIA proteomic analysis

**DOI:** 10.1186/s13020-023-00709-9

**Published:** 2023-01-28

**Authors:** Yong Chen, Xing-wang Zhu, Wing-Fu Lai, Yong-pu Liu, Xuan-feng Xu, Li-ming Liu, Yan-juan Chen, Chuan-fu Zhang, Guang-yi Wang, Zhi-qiang Cheng, Dong-zhou Liu

**Affiliations:** 1 Shenzhen People‘s Hospital (The Second Clinical Medical College of Jinan University and the First Affiliated Hospital to Southern University of Science and Technology), Shenzhen, China; 2 Shenzhen Clinical Research Centre for Geriatrics, Shenzhen People‘s Hospital, Shenzhen, China; 3 Southern Medical University Hospital of Integrated Traditional Chinese and Western Medicine, Guangzhou, China; 4 Department of Applied Biology and Chemical Technology, Hong Kong Polytechnic University, Hong Kong, Hong Kong Special Administrative Region China; 5 Department of Urology, Zhejiang Provincial People’s Hospital, Hangzhou Medical College, Hangzhou, Zhejiang China; 6 The Seventh People’s Hospital, Shanghai University of Traditional Chinese Medicine, Shanghai, China; 7 Guizhou Medical University affiliated Hospital, Guiyang, China

**Keywords:** Traditional chinese medicine, Aging, Rheumatoid arthritis, Integrative treatment, Synergic action

## Abstract

**Background:**

Elderly rheumatoid arthritis (ERA) population faces multiple treatment dilemma. Here we aim to investigate if Gancao Nourishing-*Yin* decoction (GCNY) added to methotrexate (MTX) exhibit better effects in an ERA mice model.

**Methods:**

ERA mice model was established by adding D-galactose (Dgal) to collagen-induced arthritis (CIA) mice. The model was then assigned into control group (CIA + Dgal), MTX treatment group (MTX), GCNY treatment group (GCNY), and integrative treatment group (MTX + GCNY). Pathological scoring was performed to evaluate the severity between the groups. Proteomic analysis was applied to investigate the secretory phenotype of the ERA mouse model and the underlying mechanism of GCNY, MTX and their combination. Representative cytokines related to proteomic results were further validated by ELISAs.

**Results:**

CIA + Dgal mice showed more aggressive joints damage than the CIA mice. Besides changes in the inflammatory pathway such as Pi3k-Akt signaling pathway in both model, differential expressed proteins (DEPs) indicated metabolism-related pathways were more obvious in CIA + Dgal mice. Low-dose MTX failed to show pathological improvement in CIA + Dgal mice, while GCNY improved joints damage significantly. Besides down-regulated inflammation-related targets, GCNY-regulated DEPs (such as Apoc1 ~ 3, Grk2 and Creb3l3) were broadly enriched in metabolism-related pathways. MTX + GCNY showed the best therapeutic effect, and the DEPs enriched in a variety of inflammatory,metabolism and osteoclast differentiation signaling pathway. Notably, MTX + GCNY treatment up-regulated Dhfr, Cbr1, Shmt1 involved in folic acid biosynthesis and anti-folate resistance pathways indicated a coincidence synergic action. ELISAs confirmed CPR and Akt that elevated in CIA + Dgal mice were significantly ameliorated by treatments, and adding on GCNY elevated folic acid levels and its regulator Dhfr.

**Conclusion:**

Aging aggravated joints damage in CIA, which probably due to metabolic changes rather than more severe inflammation. GCNY showed significant effects in the ERA mice model especially when integrated with MTX to obtain a synergic action.

**Supplementary Information:**

The online version contains supplementary material available at 10.1186/s13020-023-00709-9.

## Background

Elderly rheumatoid arthritis (ERA) patients are a special group of RA patients, including patients with onset after the age of 60 and the elderly with disease onset at a young age but over the age of 60 (some literatures reported 65 years as the cut-off value) [1]. Compared with young RA patients, ERA patients show the characteristics of acute onset, frequent system damage, and poorer functional prognosis [2]. ERA patients often require multiple drugs to treat various comorbidities especially age-related diseases (ARDs), and due to the decline in liver, kidney, and digestive system functions, adverse drug events and contraindications become more prominent. These factors make it difficult to optimize the complex regimen of ERA and leads to fail in ‘up to the target’ treatment [3]. Therefore, the treatment options of ERA face dilemma.

Traditional Chinese Medicine (TCM) shows promising advantages in preventing and treating aging. About 700 years ago, Zhu Danxi (AD. 1281–1358) started to disseminate his representative theory of nourishing *yin* to treat various chronic diseases. He proposed the principium that a human body is inclined to suffer from a deficiency of *yin* while *yang* is in a surplus, and this phenomenon is a shared TCM pathogenic mechanism for multiple ARDs [4]. Zhu Danxi has been regarded as one of the ‘*Four Great Masters’* of Chinese medicine. Based on this TCM principle, the TCM herbal formula, Gancao Nourishing-*Yin* decoction (GCNY), is designed to tonify *yin*, while also promoting *yang* through body regulation [5]. It improves the health status (reflected by *Qi* and *blood* of TCM) via the presence of herbal ingredients such as licorice, ginseng, jujube and ginger, and shows anti-inflammatory function to senile RA based on network pharmacology [6]. In vitro studies of GCNY-medicated serum showed inhibitory effects on proliferation of fibroblast-like synoviocytes, which are a main effector cells in RA pathogenesis [7]. Our recent study also revealed that GCNY alleviates peroxidation induced by H_2_O_2_ in HMC-3 cells [4]. Thus, GCNY is hypothesized to show potential to treat situations of aging combined with RA. However, more direct evidence in animal research is needed to confirm the effectiveness before clinical application.

This study intends to establish an ERA mouse model, which is established by adding D-galactose to the collagen-induced arthritis (CIA) model. Data-independent acquisition (data-independent acquisition, DIA) proteomic technology is applied to analyze the secretory phenotype of the ERA mouse model as compared to the CIA model, and to explore the effect and overall regulation mechanism of GCNY, and its combination application with low dose methotrexate (MTX) to the ERA model, so as to understand what integrative treatment can bring to ERA.

## Materials and methods

### Experimental animals and model establishment

SPF grade, 8-week-old (22-25 g) DBA/1 male mice were purchased from Cavens Laboratory Animal Co., Ltd. (Changzhou, China. License No. SCXK (Su) 2021-0013), and a total of 72 mice were obtained.

The experimental mice were randomly divided into three groups. The normal control group (NC, n = 10), the CIA model group (CIA, n = 12) and the group in which CIA was combined with D-galactose (CIA + Dgal, n = 50). For the NC group, 0.1 ml of glacial acetic acid (0.5 mol/L) was subcutaneously injected into the back of the mice at the time point of injection as a control for CIA injection, and 0.1 ml of distilled water was subcutaneously injected into the neck every day as a control for D-galactose injection. In the CIA group, 2 mg/ml bovine type II collagen was dissolved in 0.5 mol/L glacial acetic acid at 4 °C by stirring overnight. An equal volume of complete Freund’s adjuvant was added dropwise under an ice bath and ground into an emulsion. Subcutaneous injection of 0.1 ml of the emulsion on the back of the mice was used for the initial immunization, and 15 days later, repeat the injection as a booster immunization. In the following 30 days of observation, if the limbs and joints were swollen, the model was successfully established [8]. At the same time, 0.1 ml of distilled water was subcutaneously injected into the neck of CIA group mice every day as a control for D-galactose injection. In the CIA + Dgal group, based on the modeling method of the CIA group, D-galactose 125 mg/kg was subcutaneously injected into the neck, once a day, for 6 weeks to establish an aging mouse model [9]. The modeling measures of each group are shown in Additional file 1: Table S1. At the end of the 6th week, the mice were judged to be successful in modeling if they had brown hair, slow movement, and lethargy appearance besides of joints symptoms.

### Grouping and interventions to each group mice

The CIA + Dgal model mice were further divided into the MTX group, GCNY group and MTX + GCNY group. For, MTX group, the dosage of MTX was 1 mg/Kg, 2 times a week by gavage [10]. For the GCNY group, the herbal formula consists of Gancao (*Glycyrrhiza uralensis* Fisch.), Ginseng (*Panax ginseng* C. A. Meyer), Yuzhu (*Polygonatum odoratum* (Mill.) Druce), Luohanguo (*Momordicae grosvenori*), Pugongying (*Taraxacum mongolicum* Hand.-Mazz.), Ganjiang (dried ginger, *Zingiber officinale* Roscoe), and Dazhao (*Ziziphus zizyphus*) at ratio of 6:3:4:5:2:3:2 [4]. The flavors, meridian distributions and main function of each component were seen in Table 1. About 155 drug-ike properties were identified by TCMSP [6]. The equivalent of 3.0 g/kg of raw medicine was further concentrated for administration by gavage after boiling for 1 h, once a day. The dosage was previously proved to be effective in psoriasis-like skin inflammation lesions model mice [11]. The quality control of GCNY was conducted by high performance liquid chromatography (HPLC) as published recently [5]. MTX + GCNY group mice were treated by combining the above two interventions. CIA + Dgal group mice were treated by gavage with an equal volume of normal saline. The treatment intervention for mice is summarized in Additional file 1: Table S2.

**Table 1:** The flavors, meridian distributions and main functions of each component in GCNY

Components	Flavor	Meridian distributions	Functions	Staus
Gancao	Sweet, natured	Heart, lung, spleen, stomach	Invigorating qi, clearing heat and detoxifying	Jun (monarch)
Ginseng	Bitter, warm	Spleen, lungs, heart	Replenishing Qi, producing Jin, calming the mind	Chen (minister)
Yuzhu	Sweet, natured	Lung, spleen, stomach	Nourishing Yin, moistening dryness and quenching thirst	Chen (minister)
Pugongying	Sweet, cool	Liver and stomach	Clearing heat and detoxifying, detumescence and dispersing knot	Zuo (assistant)
Ganjiang	Acrid, heat	Spleen, stomach, kidney, heart, lung	Warming the cold, backing to Yang, warming the lung	Zuo (assistant)
Luohanguo	Sweet, cool	Lung, large intestine	Clearing the heat and moistening the lung, smoothing bowel laxity	Zuo (assistant)
Dazhao	Sweet, warm	Spleen, stomach	Tonifying qi, nourishing blood and calming the mind, and relieving the properties	Shi (guider)

### Blood and joints sample collection

After 4 weeks of treatment, blood was collected from the eyeball under general anesthesia by intramuscular injection of Zoletil (55–75 mg/kg, Virbac, France), and serum was separated by centrifugation. After overdose anesthesia, the mice were euthanized by cervical dislocation, and the lower extremity joints were obtained for pathology study.

### Symptom scoring

According to the 5-level scoring method of arthritis lesions, 2 hindlimb arthritis of mice were scored blindly, and the swelling of the feet, the incidence and conditions of hindlimb and tail lesions were observed. Specific points include: 0, no swelling; 1, slight swelling of the toe joint; 2, swelling of the toe joint and ankle; 3, swelling below the ankle; 4, swelling of all joints including the ankle [12].

### Hematoxylin-eosin (HE) staining and pathological analysis

Two hindlimb joints of each mice were taken, and the surface skin tissue was separated before soaked in an EDTA decalcification solution (SenBeiJia BioTech Co., Ltd. China) for 14 days so as to be fully decalcified, then incised along the sagittal plane of the ankle joint, followed by 4% formalin fixation, dehydration, and paraffin embedding, dewaxing, HE staining, dehydration, transparency and mounting and other conventional production processes [13].

The slides were scanned with a slide scanning image analysis system 100X (Shenzhen Shengqiang Technology Co., Ltd. China). Histological analysis was performed blindly by 2 trained pathologists using digital pathology reading software. The score included 4 pathological features, edema, synovial hyperplasia, cartilage/bone erosion, and pannus formation. Scores of 0, 1, 2, 3, and 4 were scored from none, mild, moderate, and severe in order [14]. In recent years, the important role of lipid metabolism in inflammatory response and the correlation between adipose tissue and cartilage damage have been confirmed [15], so the score of fat deposition was introduced in the current study. After synthesizing normal joints and diseased joints in each group, a 0–4 score for subcutaneous adipose tissue around the joint from none to severe was obtained by the proportion of adipose tissue.

### DIA proteomic analysis

Proteomic analysis was performed for all 6 groups of serum samples (NC, CIA, CIA + Dgal, MTX, GCNY, and MTX + GCNY group), with 4 mice serum from each group. DIA-based quantitative proteomic analysis (Shanghai Biotree Co., Ltd. China) was performed after the de-abundance process. The obtained raw data were screened for the number of unique peptides of the protein, and the number of unique peptides was ≥ 1; the missing values in the original data were simulated (Missing Value Recoding), which was the half minimum method to fill. A total of 2415 detected proteins were retained after preconditioning. The differentially expressed proteins (DEPs) were acquired by using Student’s t-test, and the Fold change was ≤ 0.83 or ≥ 1.2 and p < 0.05 [16]. Bioinformatics analysis of the proteomics data mainly include principal component analysis, KEGG pathway analysis, and Mfuzz cluster analysis.

### Enzyme-linked immunosorbent assays (ELISAs)

Serum samples from each group were centrifugated under 1000 g for 20 min and supernatant were then attenuanted by sample diluent provided in ELISA kits. Targets of C-reactive protein (CRP), Protein kinase B (Akt), Dihydrofolate reductase (Dhfr) and folic acid were measured by commercially available ELISA kits (Jianglai Bio Co., Ltd, China) according to the manufacturer’s protocols. The ELx808 absorbance microplate reader was used to read the optical density (OD) value at 450 nm. The concentrations of the samples were calculated based on the standard curve.

### Statistical analysis

The arthritis scores, pathological scores and CRP, Akt, Dhfr, folic acid levels examined by ELISAs of mice in each group were statistically analyzed using GraphPad Prism 9 software. The data were expressed as mean ± standard deviation, using analysis of variance or t test to compare data with normal distribution and nonparametric test to analyze non-normal distribution data; p < 0.05 means the difference is statistically significant. Representative DEPs screened out by DIA proteomics were also presented with interleaved bars of GraphPad Prism, with the normalized response intensity of peptide ions on the mass spectrometer being used as the relative expression level.

## Results

### The aging CIA mouse model (CIA + dgal group) showed more severe joint damage

**Fig. 1:**
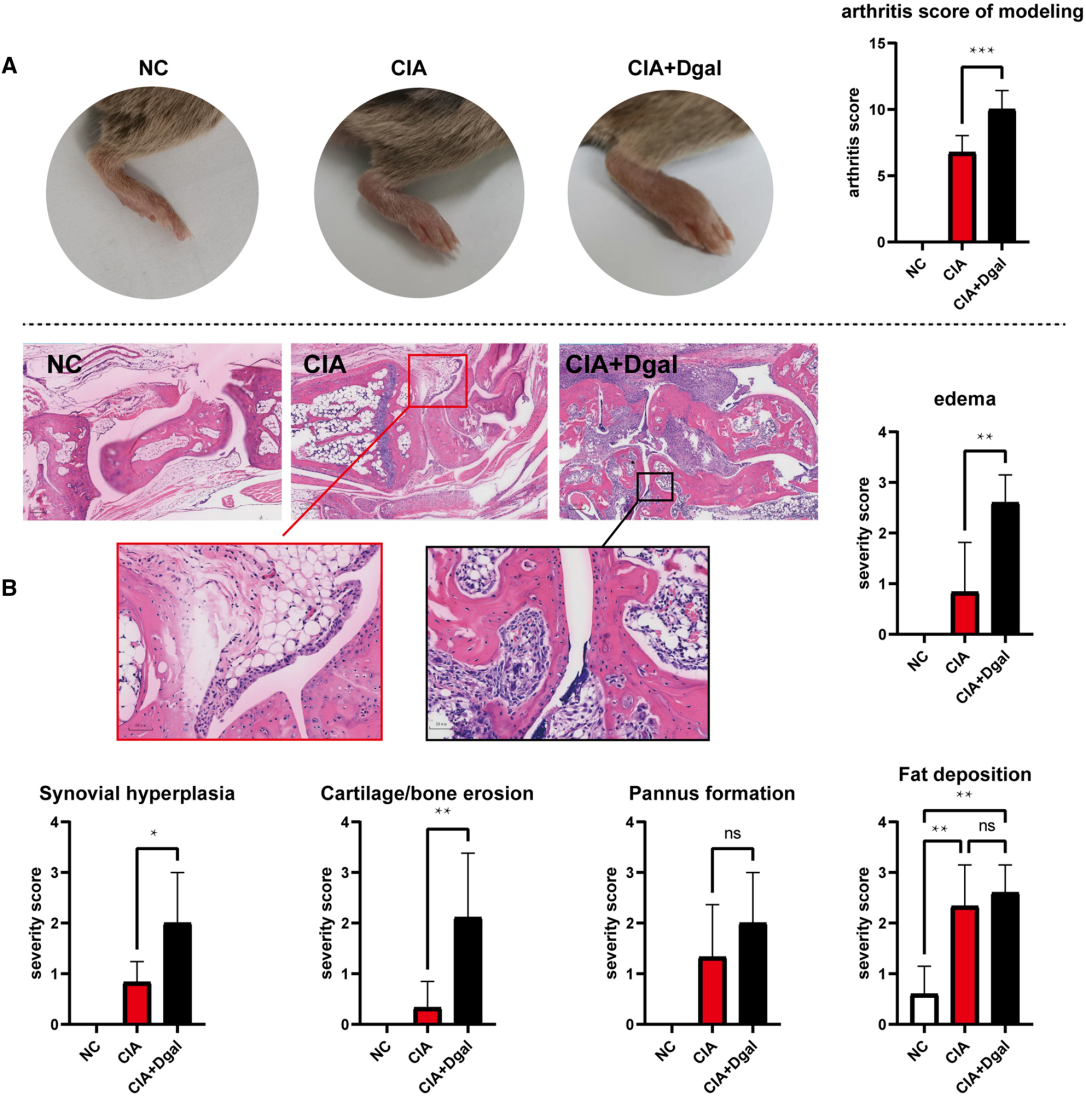
Arthritis symptoms and pathological scores of mice in each group. **A** Clinical symptoms and arthritis score of NC, CIA and CIA + Dgal mice; **B** Ankle H-E staining and severity score on edema, synovial hyperplasia, cartilage/bone erosion, pannus formation and fat deposition in NC, CIA and CIA + Dgal group (magnification of microscope, 4 × 2; local magnification 4 × 10 times). *ns* no significance, *P<0.05, **P < 0.01

Of the total of 62 mice that underwent CIA modeling, 49 (79%) showed typical arthritis onset. In the end, there were 10 mice in the NC group, 9 mice in the CIA group, 9 mice in the CIA + Dgal group, 11 mice in the MTX group, 9 mice in the GCNY group, and 11 mice in the MTX + GCNY group. Arthritis scores in CIA + Dgal mice showed more severe clinical symptoms (Fig. 1A). The pathological scores showed that the pathological features of hindlimb joint edema, synovial hyperplasia, and cartilage and bone erosion in CIA + Dgal mice were significantly higher than those in the pure CIA model, and the scores of pannus formation and fat deposition were also slightly higher, though without showing statistical significance (Fig. 1B).

### The secretory phenotype of CIA + dgal mice is different from that of pure CIA mice

**Fig. 2:**
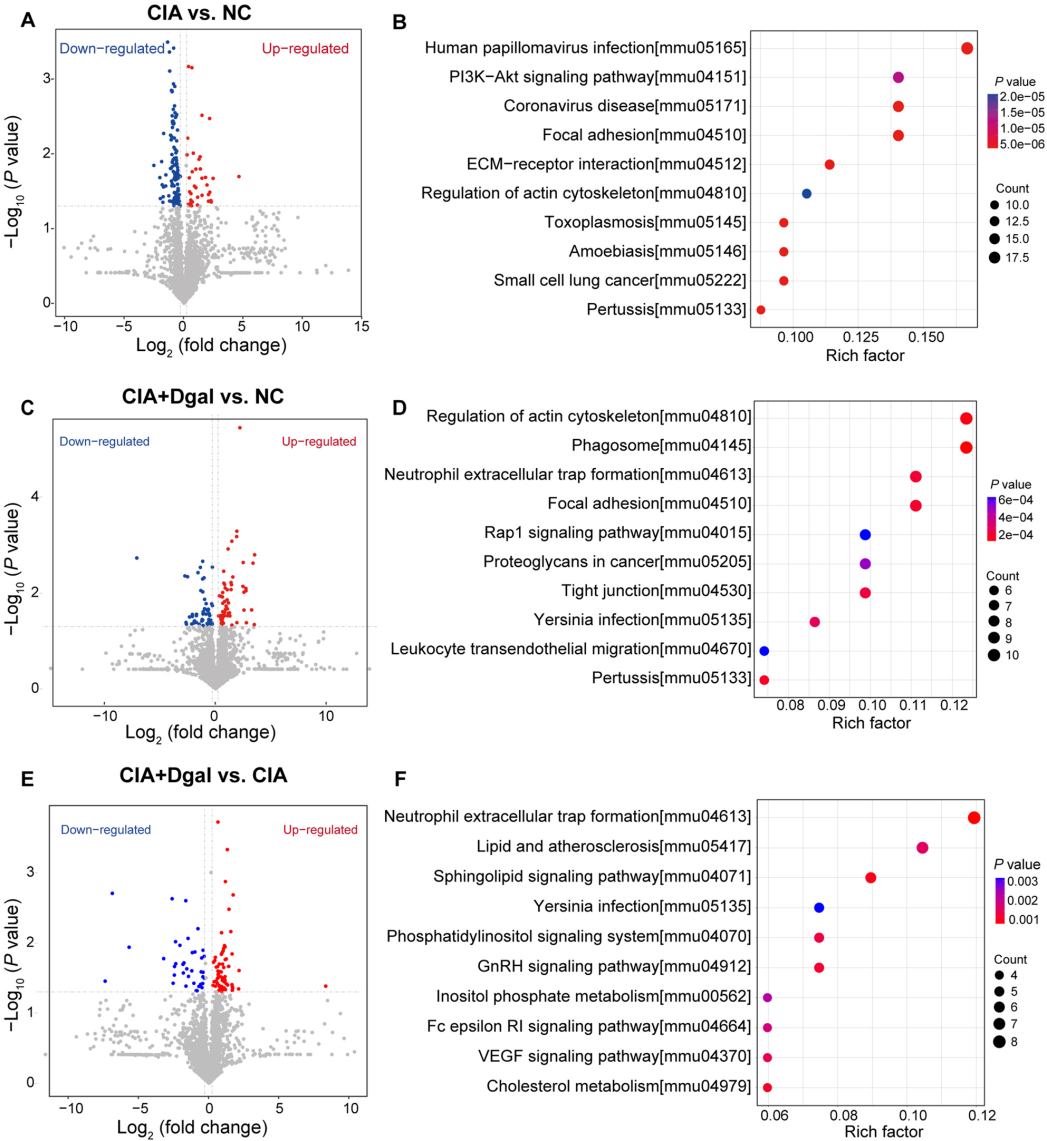
Differences in secretory phenotypes between NC, CIA and CIA + Dgal mice. **A**, **B** DEPs between NC and CIA mice and their KEGG enrichment analysis. **C**, **D** DEPs between NC and CIA + Dgal mice and their KEGG enrichment analysis. **E**, **F** DEPs between CIA + Dgal and CIA mice and their KEGG enrichment analysis

Through proteomic study, 88 proteins were up-regulated and 120 proteins were down-regulated in the CIA group compared with the NC group (Fig. 2A). These DEPs are mainly enriched in human papillomavirus infection, PI3K-Akt signaling pathway, coronavirus disease, IL-17 signaling pathway and TNF signaling pathway through KEGG analysis (Fig. 2B and Additional file 2). Compared with NC mice, the CIA + Dgal group showed 77 up-regulated proteins and 67 down-regulated proteins (Fig. 2C), mainly enriched in regulation of actin cytoskeleton, phagosome, neutrophil extracellular trap formation, focal adhesion, Rap1 signaling pathway, human papillomavirus infection, PI3K-Akt signaling pathway (Fig. 2D and Additional file 3).

It can be seen that the change of metabolism is an important feature of the secretory phenotype of CIA + Dgal group mice. By directly comparing the CIA + Dgal group and CIA mice, there were 71 up-regulated proteins and 70 down-regulated proteins (Fig. 2E), mainly enriched in neutrophil extracellular trap formation, lipid and atherosclerosis, and sphingolipid signaling pathway etc. (Fig. 2F and Additional file 4).

**Fig. 3:**
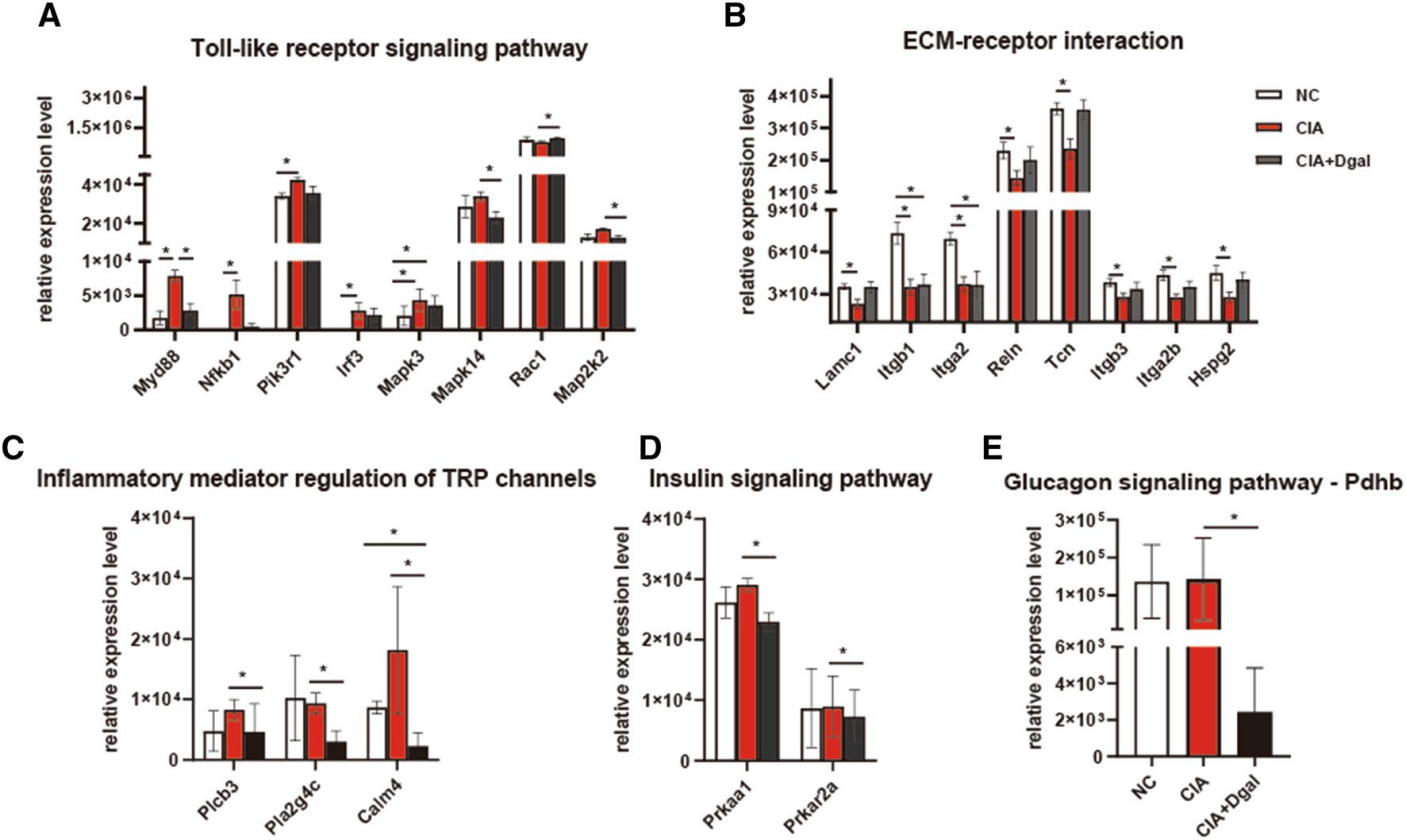
Representative DEPs of CIA and CIA + Dgal model mice. **A** Changes of Toll-like receptor signaling pathway related targets in CIA and CIA + Dgal groups; **B** CIA group down-regulated multiple targets of ECM receptor interaction pathway; **C** Compared with the CIA group, the CIA + Dgal group showed lower expression of related targets in inflammatory mediated transient receptor potential (TRP) ion channels; **D–E** Compared with CIA group, CIA + Dgal group down-regulated insulin signaling pathway and glycogen signaling pathway related targets. * P < 0.05

**Fig. 4:**
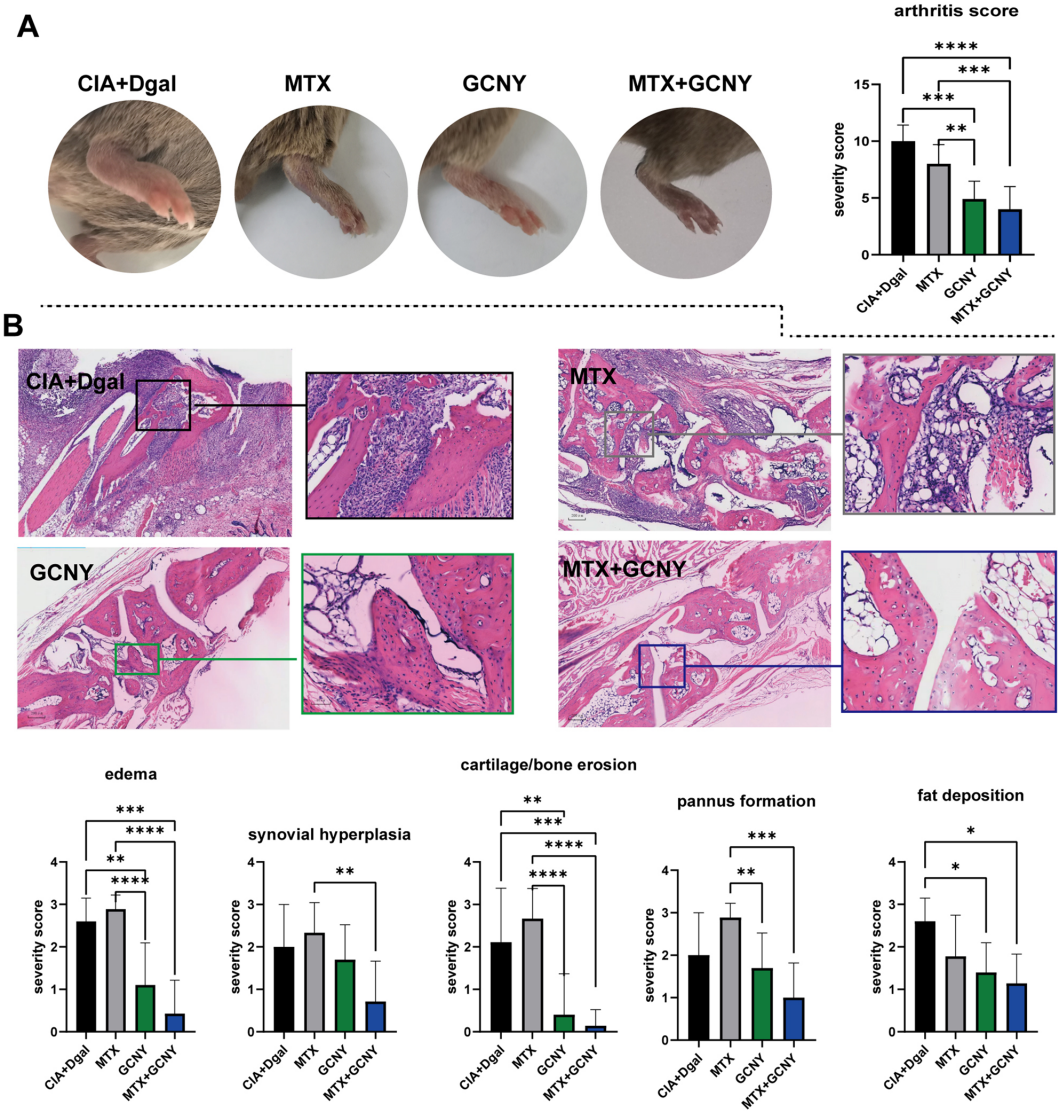
Arthritis symptoms and pathological scores of mice in CIA + Dgal, MTX, GCNY and MTX + GCNY groups. **A** Clinical symptoms and arthritis score of CIA + Dgal, MTX, GCNY and MTX + GCNY groups. **B** Ankle H-E staining and severity score on edema, synovial hyperplasia, cartilage/bone erosion, pannus formation and fat deposition in CIA + Dgal, MTX, GCNY and MTX + GCNY groups. (magnification of microscope, 4 × 2; local magnification 4 × 10 times). *ns* no significance, *P < 0.05, **P < 0.01, ***P < 0.01, ****P < 0.0001

In Toll-like receptor signaling pathway, Myd88, Nfkb1, Pik3r1, Irf3, and Mapk3 in the CIA group were higher than those in the NC group; Mapk14 and Map2k2 in the CIA + Dgal group were lower than those in the CIA group, and Rac1 was higher in the CIA + Dgal group (Fig. 3A and Additional file 1: Figure S1). In the CIA group, compared to NC mice, Lamc1, Itgb1, Itga2, Reln, Tcn, Itgb3, Itga2b and Hspg2 were all down-regulated in the extracellular matrix receptor interaction pathway (Fig. 3B Additional file 1: Figure S2). Compared with the CIA group, the Plcb3, Pla2g4c and Calm4 were down-regulated in the CIA + Dgal group on inflammatory mediator regulation of TRP channels (Fig. 3C Additional file 1: Figure S3). In gonadotropin-releasing hormone (GnRH) signaling pathway, targets of Mapk14, Plcb3, Map2k2, Pla2g4c and Calm4 and in insulin signaling pathway targets of Prkar2a, Prkaa1, Map2k2, Calm4 were also down-regulated (Fig. 3A, C, D and Additional file 1: Figure S4). Glycogen signaling pathway targets of Plcb3, Prkaa1, Pdhb, Calm4 were also down-regulated in the CIA + Dgal group (Fig. 4C, D, E and Additional file 1: Figure S5) It can be seen that the secretion phenotype of the CIA + Dgal group and the pure CIA group have some of the same inflammatory pathway changes, but there are also obvious differences, especially targets down-regulated on metabolism seen in CIA + Dgal mice.

### Low-dose MTX has no obvious clinical effect, but showed improvement in molecular biology

**Fig. 5:**
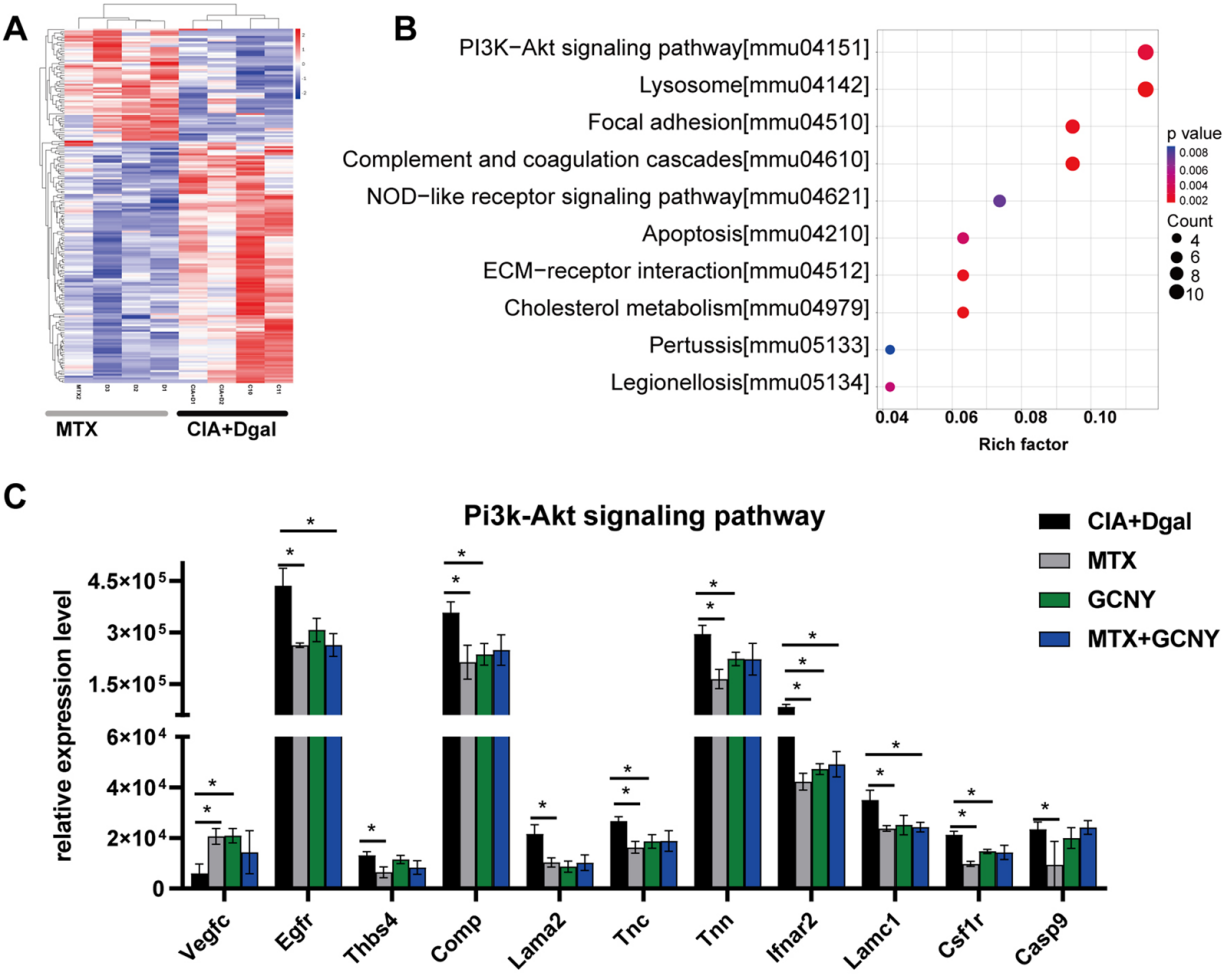
DEPs of CIA + Dgal mouse model treated with MTX. **A**, **B** DEPs: MTX group vs. CIA + Dgal group, heat map and KEGG analysis. **C** Effects of MTX, GCNY and MTX + GCNY on other targets of PI3K-AKT signaling pathway. * P < 0.05

The CIA + Dgal mice were treated with MTX, the preferred drug for clinical ERA [17], but it did not show significant efficacy as demonstrated in clinical arthritis scores and pathological scores (Fig. 4A and B). Through proteomic analysis, MTX treatment resulted in up-regulation of 62 proteins and down-regulation of 131 proteins in CIA + Dgal model mice (Fig. 5A), which were mainly enriched in PI3K-Akt signaling pathway, lysosome, complement and coagulation cascade (Fig. 5B and Additional file 5). Thus, MTX showed a regulatory effect on inflammation and metabolism in CIA + Dgal mice. For example, in the PI3K-Akt signaling pathway, in addition to the up-regulation of Vegfc and Egfr, there are 9 targets (including Ifnar2, Lamc1, Csf1r, Lama2, Tnc, Tnn, Casp9, Comp, and Thbs4) that were significantly down-regulated (Fig. 5C).

### The mechanism by which GCNY interferes with aging CIA

**Fig. 6:**
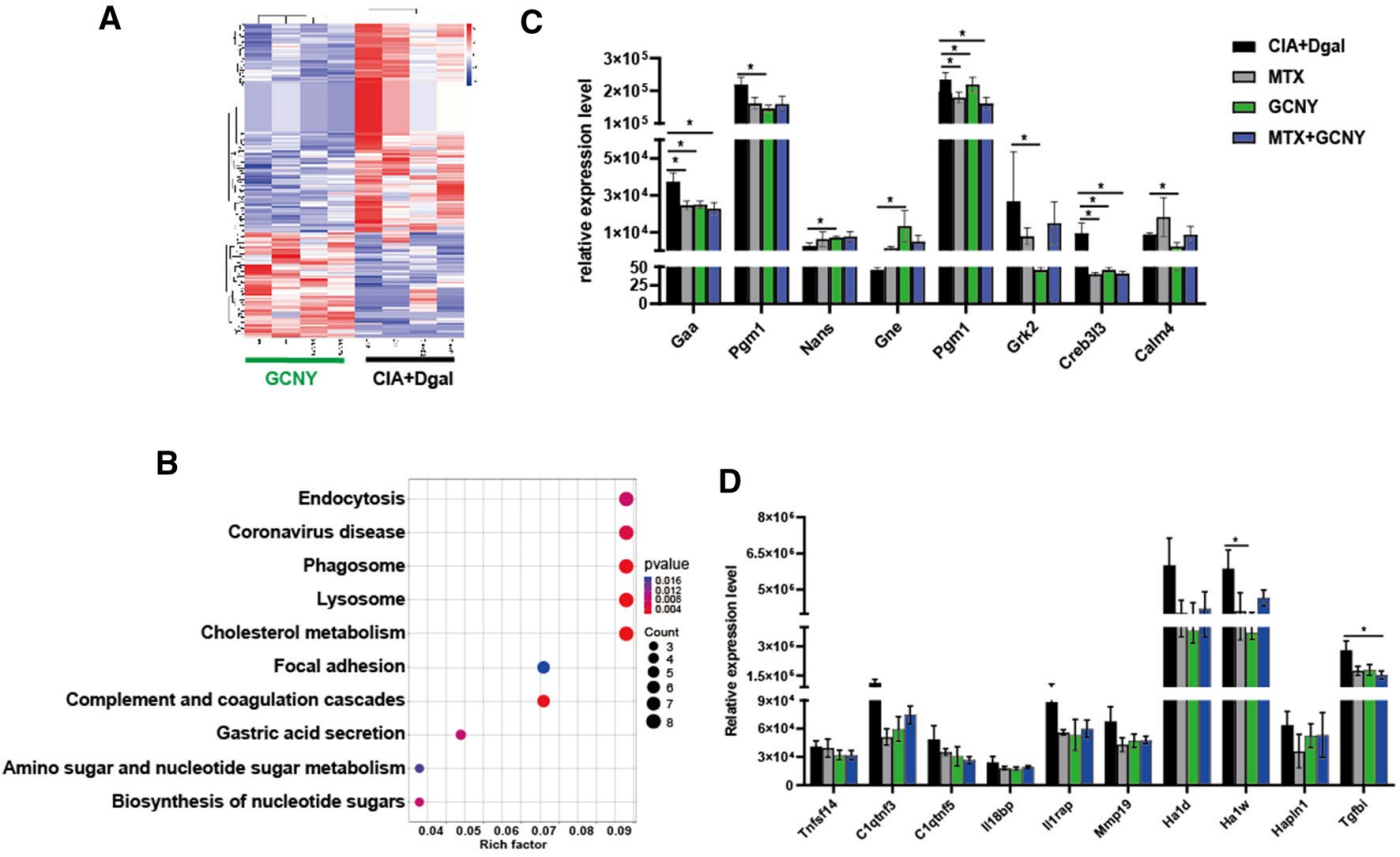
Molecular biological mechanism of GCNY acting on CIA + Dgal mouse model. **A**, **B** DEPs and KEGG pathway analysis for GCNY vs. CIA + Dgal group. **C** GCNY down-regulates the related targets of metabolism pathway in the CIA + Dgal model. **D** MTX, GCNY and MTX + GCNY shows inhibitory effects on multiple inflammatory targets. * P < 0.05

The GCNY intervention showed therapeutic effects on CIA + Dgal mice in terms of arthritis score and pathological score (Fig. 4A and B). Proteomic analysis showed that GCNY up-regulated 65 targets and down-regulated 132 targets in CIA + Dgal mice (Fig. 6A), mainly enriched in lysosome, complement and coagulation cascade, phagosome, coronavirus disease, lipid metabolism pathways (Fig. 6B). It can be seen that the effect of GCNY on the inhibition of inflammation and the effect on cells is similar to that of MTX, such as the effect on the inhibitory trend of PI3K-Akt signaling pathway (Fig. 5C).

In addition, GCNY intervention targets are more inclined to metabolism-related pathways, such as nucleotide sugar biosynthesis, amino sugar and nucleotide sugar metabolism, vitamin digestion and absorption, galactose metabolism, starch and sucrose metabolism, etc. (Additional file 6). For example, targets such as Apoa4, Apoc3, Apoc1, Pltp, Apoa1, Apoc2, Sort1, and Pcsk9 in the cholesterol metabolism pathway were all down-regulated (Additional file 1: Figure S6). The metabolic pathway-related targets including Gaa, Pgm1, Grk2, Creb3l3, and Calm4 were significantly down-regulated while Gne and Nans were up-regulated (Fig. 6D). Other inflammatory factors (such as Stat1, Tnfsf14, C1qtnf3, C1qtnf5, Il18bp, Il1rap, Mmp19, Ha1d, Ha1w, Hapln1 and Tgfbi) tended to be down-regulated (mostly faild to show statistical significance) in GCNY and MTX, or combining the two treatment modalities together. (Fig. 6E).

### GCNY combined with low-dose MTX can achieve the best curative effect for aging CIA mice

**Fig. 7:**
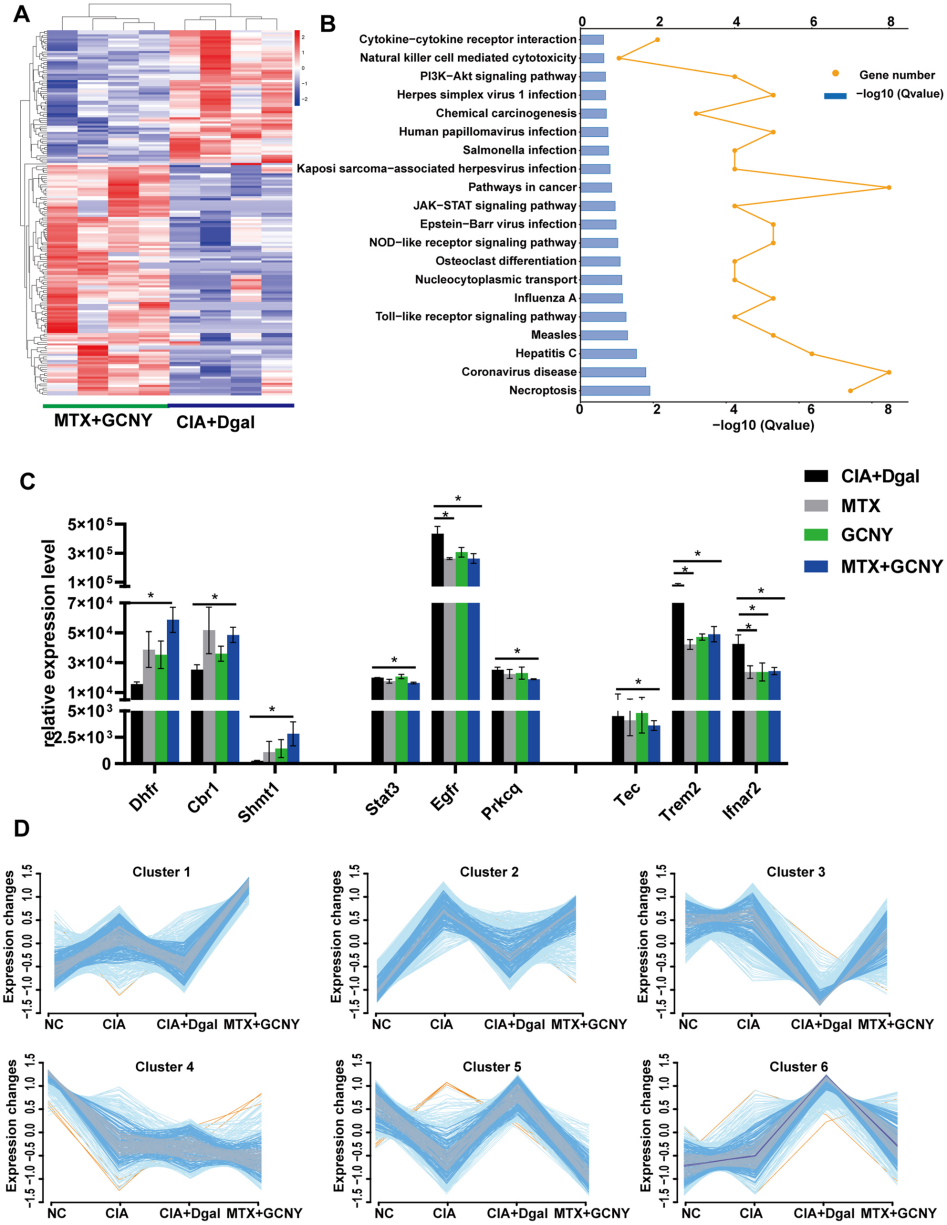
Molecular biological mechanism of MTX + GCNY treatment on CIA + Dgal mouse model. **A**, **B** DEPs and KEGG pathway analysis in MTX + GCNY vs. CIA + Dgal group. **C** Representative DEPs of CIA + Dgal, MTX, GCNY and MTX + GCNY group. **D** Mfuzz cluster analysis was performed on NC, CIA, CIA + Dgal and MTX + GCNY groups protemics. *P < 0.05

According to arthritis score and pathological feature score, MTX combined with GCNY showed the best therapeutic effect than MTX or GCNY alone (Fig. 4A and B). DIA proteomics showed that MTX + GCNY treatment up-regulated 111 proteins and down-regulated 65 proteins in model mice (Fig. 7A), mainly enriched in necroptosis, coronavirus disease, and a variety of inflammatory condition-related pathways such as JAK-STAT signaling pathway, and PI3K-Akt signaling pathway (Fig. 7B and Additional file 7). Among them, Dhfr, Cbr1, Shmt1 involved in folic acid biosynthesis and antifolate resistance pathways are all up-regulated (Fig. 7C); Stat1, Stat3, Egfr and Prkcq (which involved in PD-1 checkpoint pathway and various inflammatory pathways) were significantly down-regulated; Tec, Trem2, Ifnar2 and Stat1 involved in osteoclast differentiation and were significantly down-regulated (Figs. 6E and 7C).

Mfuzz cluster analysis was performed on the proteins of the NC, CIA, CIA + Dgal, MTX + GCNY groups, and 6 groups with changing trends of relatively independent protein clusters were found. The changes in clusters 1, 2, 3, 5, and 6 are all in line with the principle of allopathic therapy, especially the proteins in cluster 6, in which the proteins of the CIA + Dgal group were higher than the CIA group and the NC group in turn, but the MTX + GCNY treatment made them tended to be normal (Fig. 7D).

### Validate DIA proteomics results by ELISA

**Fig. 8:**
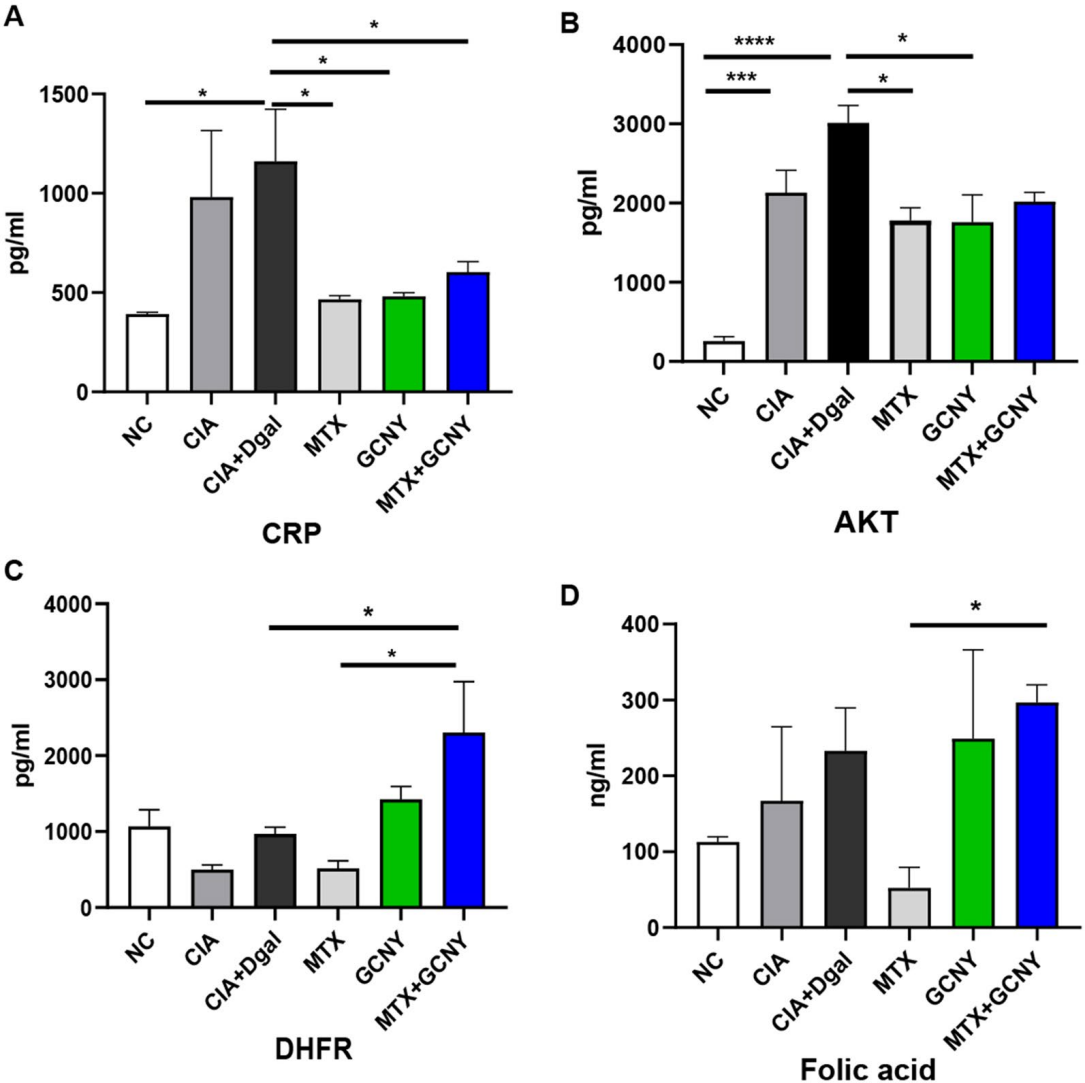
Validation of results by cytokines. **A**, **B** CRP and Akt levels were most significantly promoted in CIA + Dgal group mice, and both significantly decreased after MTX or GCNY treatment. **C**, **D** By adding GCNY to MTX, the synthesis of folic acid were rescued by its regulator Dhfr in MTX + GCNY treated mice than MTX treatment

CRP is routinely assessed as a marker of systemic inflammation and RA as well, and it is also lniked to implications such as atherosclerosis and metabolic syndrome [18]. In the current study, CRP in CIA + Dgal mice showed most significant elevation, and all treatments of MTX, GCNY and MTX + GCNY decreased serum CRP levels (Fig. 8A). Likewise, Akt, as a core target in Pi3k-Akt signaling pathway contributing to RA development [19], was verified to be significantly promoted in CIA and CIA + Dgal groups by DIA ptoteomic approach and ELISAs. Both MTX and GCNY significantly decreased Akt levels in CIA + Dgal mice (Fig. 8B). Dihydrofolate reductase (Dhfr) deficiency has been linked to megaloblastic anemia and main side effects of MTX treatment [20]. These side effects were proved to be rescued by adding GCNY to MTX (Fig. 8C and D).

## Discussion

With an increase in human life expectancy, the population of ERA expands significantly. ERA patients have clinical and biological characteristics totally different from the young counterparts [21]. Some scholars believe that the fail treat-to-target in ERA is related to the lack of proactive clinical treatment, while at the same, RA treatment guidelines or expert opinion emphasize that biological agents do not increase the adverse events in the ERA population [22, 23]. However, geriatric features do exist in the ERA population, and this is an objective reason to exlain clinicians’ treatment scruples. Therefore, strengthening research in this field is of practical significance.

There is no report of establishing ERA animal models so far. In this study, the use of the classical aging model establishment method, combined with the CIA mouse model, to successfully establish the ERA model is an innovative approach to establish animal models. It is reported that with an increasing age, the enhancement of the innate immune system activates chronic inflammatory responses and increases the incidence of ARDs; while acquired immune function deficiency can lead to impairment of immune tolerance and aggravate autoimmune responses [24]. The CIA + Dgal model established in this study confirms that aging leads to more severe joint damage, and more obviously elevated serum CRP levels, but through our primary proteomic research, it seems that the aggravation of joint damage is not necessarily due to higher levels of inflammation. In fact, inflammation-mediated transient receptor potential (TRP) ion channel, Toll-like receptor signaling pathway and their related targets were not significantly increased when compared with those in the pure CIA model. However, changes in metabolism such as, DEPs enriched in KEGG pathways of lipid and atherosclerosis, sphingolipid signaling, insulin signaling, and glycogen signaling seem to be more predisposed in the CIA + Dgal model. The evidence for the effects of aging on bone-joint metabolism is profoundly investigated [25]. This may be a more proper mechanism explaining that the ERA population faces more severe joint damage in the clinic. Also, based on DIA proteomics analysis, it seems that this ERA model successfully replicated disorders of aging and RA. The in-depth similarities between this type of model with real clinical patients need to be further investigated, including function of various organs (e.g., liver, and kidney) and comorbidities such as atherosclerosis, diabetic milieus, nerve degeneration.

After the successful establishment of the ERA model, MTX alone did not show significant efficacy. This may be because we used a comparatively small dose [26], but it satisfies the conversion relationship between mice and RA patients (10–20 mg/week based on 60 kg for human adults), which is 12.3 times that of humans. Under the intervention of MTX, PI3K-Akt and other inflammatory signaling pathway-related targets were significantly down-regulated. GCNY alone can significantly reduce the joint damage in the ERA model, and it shows a certain down-regulation trend for many inflammatory targets, among which Ha1w and Ha1d (both encoded by the H2-K1 gene, equivalent to class I histocompatibility antigens) are down-regulated. TNF family proteins (Tnfsf14, C1qtnf3, C1qtnf5), interleukin family (Il18bp, Il1rap), Mmp19, Hapln1, and Tgfbi were all down-regulated to varying degrees by MTX or GCNY alone. Among them, Hapln1 is our newly identified target that promotes the inflammatory phenotype of RA [13]. GCNY has obvious regulatory effects on many metabolic pathways, such as lipid metabolism pathway, galactose metabolism pathway and their targets were significantly down-regulated. Positive synthesis of lipid metabolism is not only the characteristics of aging [27, 28], but can also promote the development of inflammation [29, 30]. The down-regulation of these targets by GCNY reflects its inhibitory effect on both inflammation and aging.

Although low-dose MTX does not appear to have significant clinical efficacy, it appears to be most effective when combined with GCNY. The inhibition of various inflammation factors is also the most significant, such as Stat1, Hspb6 and Tgfb. Pi3k-Akt signaling pathway has been profoudly investigated within RA by promoting angiogenesis and activation of fibroblast-like synoviocytes, and testified as reliable therapeutic target [19, 31, 32]. Besides, it participates in aging and ARDs by crosstalk with oxidative stress, DNA damage, apoptosis and inflammation [33, 34]. Here in the study, Pi3k-Akt signaling pathway-related targets were up-regulated in CIA and ERA model mice, while significantly down-regulated by treatment of MTX, GCNY or integrated. It is worth noticing that targets of Dhfr, Cbr1 and Shmt1 that related to folic acid biosynthesis were up-regulated. MTX exerted anti-rheumatic and anti-tumor effects by inhibiting cellular protein synthesis and inhibiting cell division [26, 35], which is also the reason why MTX leads to side effects such as anemia. Clinically, this side effect is often corrected by supplementing folic acid [36]. It is interesting that the combination of GCNY and MTX potentially exempts receivers from the loss of folate. Besides the benefit of folic acid metabolism, the combination of MTX + GCNY also significantly inhibited the related targets of the osteoclast differentiation pathway, suggesting that the combination therapy has a superior regulatory effect on bone metabolism.

## Conclusion

DIA proteomics adopted in this study is a mass spectrometry analysis strategy developed in recent years, which has the advantages of broad protein coverage, high reproducibility and high accuracy [37]. On the basis of histological analysis and considering the secretory phenotype of DIA proteomics in the ERA mouse model, a theory that is different from the view of “aging aggravates inflammation in RA” has emerged. In another word, it is not aging that promotes inflammation in RA patients. It is more likely that more severe joint destruction of the ERA is caused by metabolic alterations. And through this study, the attenuating and synergistic effects of GCNY and MTX in the treatment of ERA are well reflected, and this is worthy of further in-depth clinical research.

## Supplementary Information

**Additional file 1: Table S1.** The modeling measures of each group. **Table S2.** The treatment intervention for CIA + Dgal mice. **Figure S1. **Compare to NC group, CIA group mice up-regulatedDEPs in Toll-like receptor signaling pathway, such as, Myd88, Nfkb1, Pik3r1,Irf3, and Mapk3. The up-regulated DEPs are highlighted in red. **Figure S2. **Compare to NC group, CIA group micedown-regulated DEPs in extracellular matrix receptor interaction pathway, such as,Lamc1, Itgb1, Itga2, Reln, Tcn, Itgb3, Itga2b and Hspg2. The down-regulatedDEPs are highlighted in blue. **Figure S3. **Compared with the CIA group, the Plcb3, Pla2g4cand Calm4 were down-regulated in the CIA+Dgal group on Inflammatory mediatorregulation of TRP channels. The down-regulated DEPs are highlighted in blue. **Figure S4. **Compared with the CIA group, (A) Mapk14, Plcb3,Map2k2, Pla2g4c and Calm4 in gonadotropin-releasinghormone (GnRH) signaling pathway and (B) targets of Prkar2a, Prkaa1, Map2k2 andCalm4 in insulin signaling pathway are down-regulated in the CIA+Dgal group.The down-regulated DEPs are highlighted in blue and typed out aside. **Figure S5.** Compared with the CIA group, Plcb3, Prkaa1, Pdhband Calm4 in Glycogen signaling pathway are down-regulated in the CIA+Dgalgroup. The down-regulated DEPs are highlighted in blue and typed out aside. **Figure S6.** GCNY down-regulated targets of lipid metabolismpathway in CIA+Dgal model. The down-regulated DEPs are highlighted in blue andtyped out aside.

**Additional file 2.** DEPs and their enriched KEGG pathways of CIA vs. NC mice.

**Additional file 3.** DEPs and their enriched KEGG pathways of CIA + Dgal vs. NC mice.

**Additional file 4.** DEPs and their enriched KEGG pathways of CIA + Dgal vs. CIA mice.

**Additional file 5.** DEPs and their enriched KEGG pathways of MTX vs. CIA + Dgal mice.

**Additional file 6.** DEPs and their enriched KEGG pathways of GCNY vs. CIA + Dgal mice.

**Additional file 7.** DEPs and their enriched KEGG pathways of MTX + GCNY vs. CIA + Dgal mice.

## Data Availability

The original contributions presented in the study are included in the article/Supplementary Material. Further inquiries can be directed to the corresponding author.

## Abbreviations

RA: Rheumatoid arthritis
Dgal: D-galactose
CIA: Collagen induced arthritis
ARDs: Age related diseases
GCNY: Gancao Nurishing-*yin*decoction
DEPs: Differentially expressed proteins
CRP: C-reactive protein
