# Supplementary material for: Gancao Nourishing-Yin decoction combined with methotrexate in treatment of aging CIA mice: a study based on DIA proteomic analysis
Source: Chin Med. 2023 Jan 28;18:9. doi: 10.1186/s13020-023-00709-9 (PMC9883815; doi:10.1186/s13020-023-00709-9)
Supplement: Supplementary file 1 — Additional file 1: Table S1. The modeling measures of each group. Table S2. The treatment intervention for CIA + Dgal mice. Figure S1. Compare to NC group, CIA group mice up-regulatedDEPs in Toll-like receptor signaling pathway, such as, Myd88, Nfkb1, Pik3r1,Irf3, and Mapk3. The up-regulated DEPs are highlighted in red. Figure S2. Compare to NC group, CIA group micedown-regulated DEPs in extracellular matrix receptor interaction pathway, such as,Lamc1, Itgb1, Itga2, Reln, Tcn, Itgb3, Itga2b and Hspg2. The down-regulatedDEPs are highlighted in blue. Figure S3. Compared with the CIA group, the Plcb3, Pla2g4cand Calm4 were down-regulated in the CIA+Dgal group on Inflammatory mediatorregulation of TRP channels. The down-regulated DEPs are highlighted in blue. Figure S4. Compared with the CIA group, (A) Mapk14, Plcb3,Map2k2, Pla2g4c and Calm4 in gonadotropin-releasinghormone (GnRH) signaling pathway and (B) targets of Prkar2a, Prkaa1, Map2k2 andCalm4 in insulin signaling pathway are down-regulated in the CIA+Dgal group.The down-regulated DEPs are highlighted in blue and typed out aside. Figure S5. Compared with the CIA group, Plcb3, Prkaa1, Pdhband Calm4 in Glycogen signaling pathway are down-regulated in the CIA+Dgalgroup. The down-regulated DEPs are highlighted in blue and typed out aside. Figure S6. GCNY down-regulated targets of lipid metabolismpathway in CIA+Dgal model. The down-regulated DEPs are highlighted in blue andtyped out aside. [file 13020_2023_709_MOESM1_ESM.docx]

Additional file

# Additional file tables

Additional file: **Table 1.** The modeling measures of each group.

| Group | Acetic acid | Type II collagen + complete Freund's adjuvant | Distilled water | D-galactose |
| --- | --- | --- | --- | --- |
| NC | + | - | + | - |
| CIA | - | + | + | - |
| CIA+Dgal | - | + | - | _+_ |

Acetic acid, given subcutaneous injection is a placebo for Type II collagen + complete Freund's adjuvant injection. Distilled water, subcutaneous injection is a placebo for D-galactose injection.

NC, normal control group. CIA, collagen induced arthritis group. CIA + Dgal, CIA and D-galactose injection group.

Additional file: **Table 2.** The treatment intervention for CIA + Dgal mice.

| Group | NS | MTX medicine | GCNY  medicine |
| --- | --- | --- | --- |
| CIA+Dgal | + | - | - |
| MTX (group) | - | + | - |
| GCNY (group) | - | - | + |
| MTX+GCNY | - | + | + |

NS, normal saline administered intragastrically as placebo for MTX and GCNY. MTX, Methotrexate. GCNY, Gancao Nurishing-yin decoction.

1. Additional file **figures**


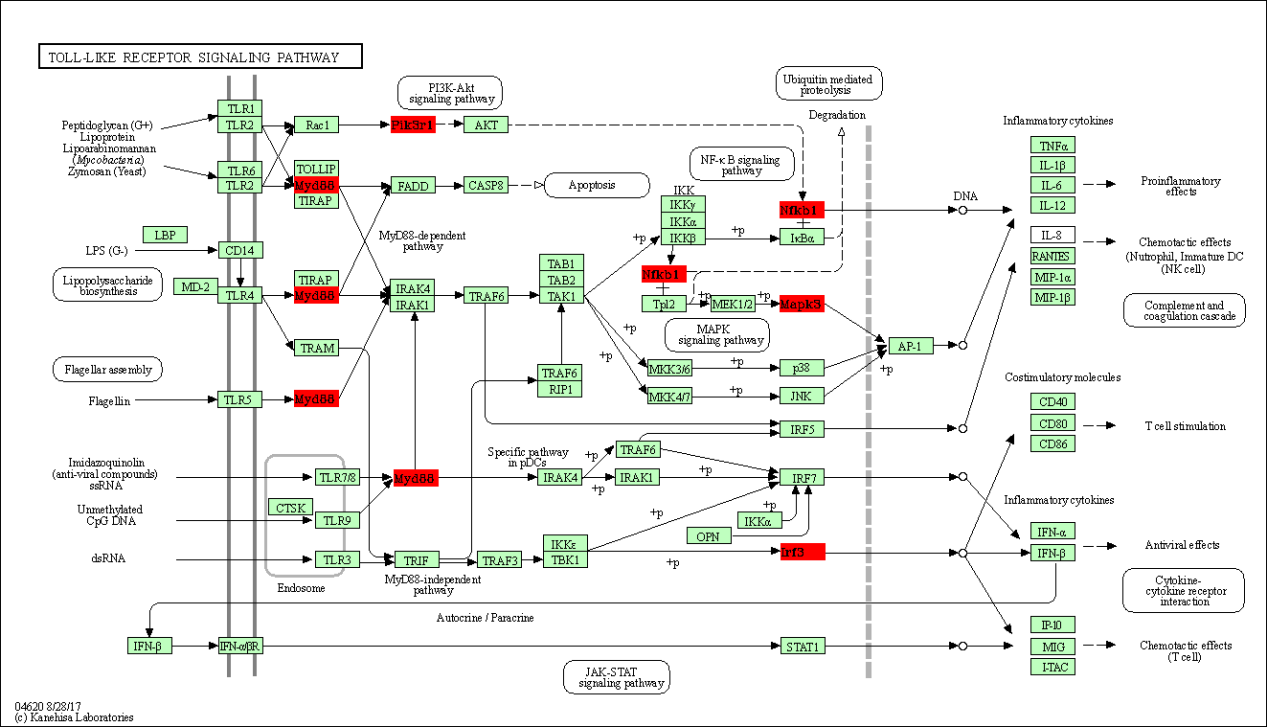


Additional file **:figure 1.** Compare to NC group, CIA group mice up-regulated DEPs in Toll-like receptor signaling pathway, such as, Myd88, Nfkb1, Pik3r1, Irf3, and Mapk3. The up-regulated DEPs are highlighted in red.


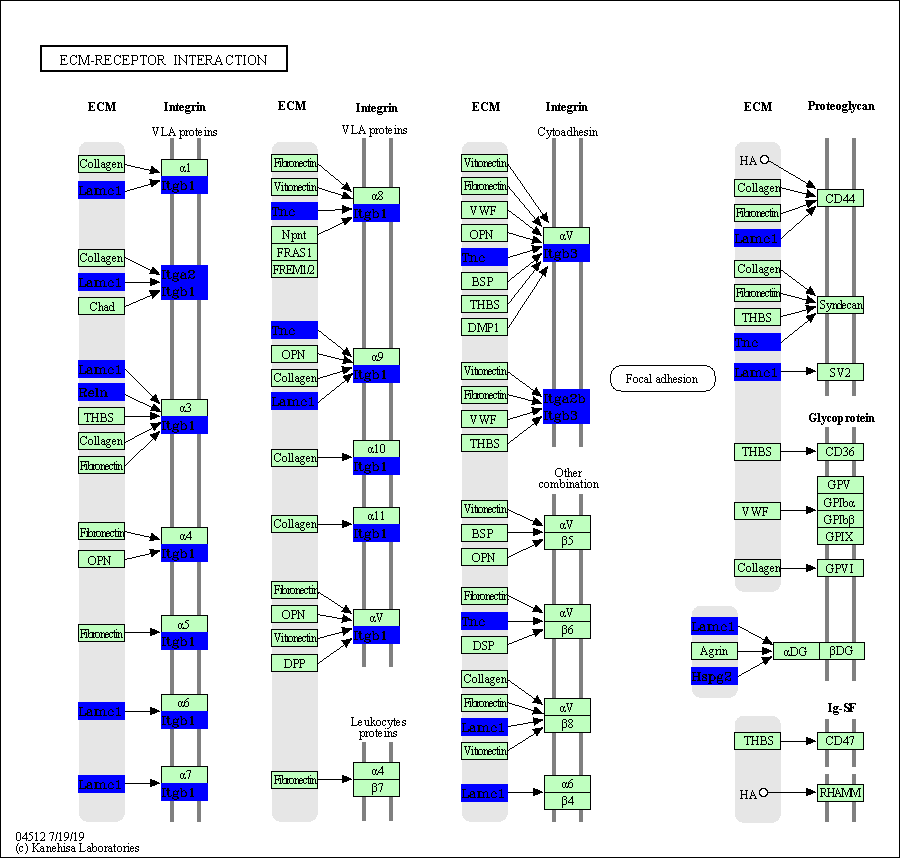


Additional file: **figure 2.** Compare to NC group, CIA group mice down-regulated DEPs in extracellular matrix receptor interaction pathway, such as, Lamc1, Itgb1, Itga2, Reln, Tcn, Itgb3, Itga2b and Hspg2. The down-regulated DEPs are highlighted in blue.


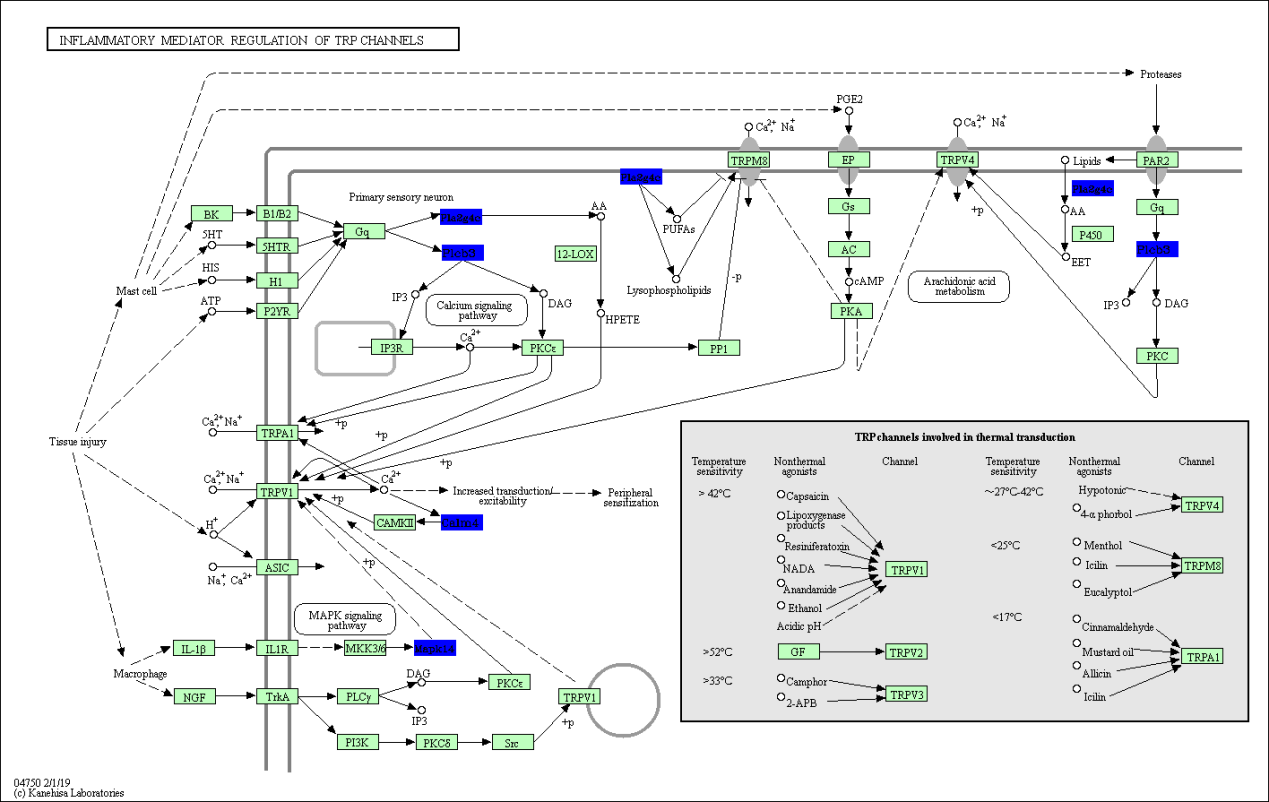


Additional file: **figure 3.** Compared with the CIA group, the Plcb3, Pla2g4c and Calm4 were down-regulated in the CIA+Dgal group on Inflammatory mediator regulation of TRP channels. The down-regulated DEPs are highlighted in blue.


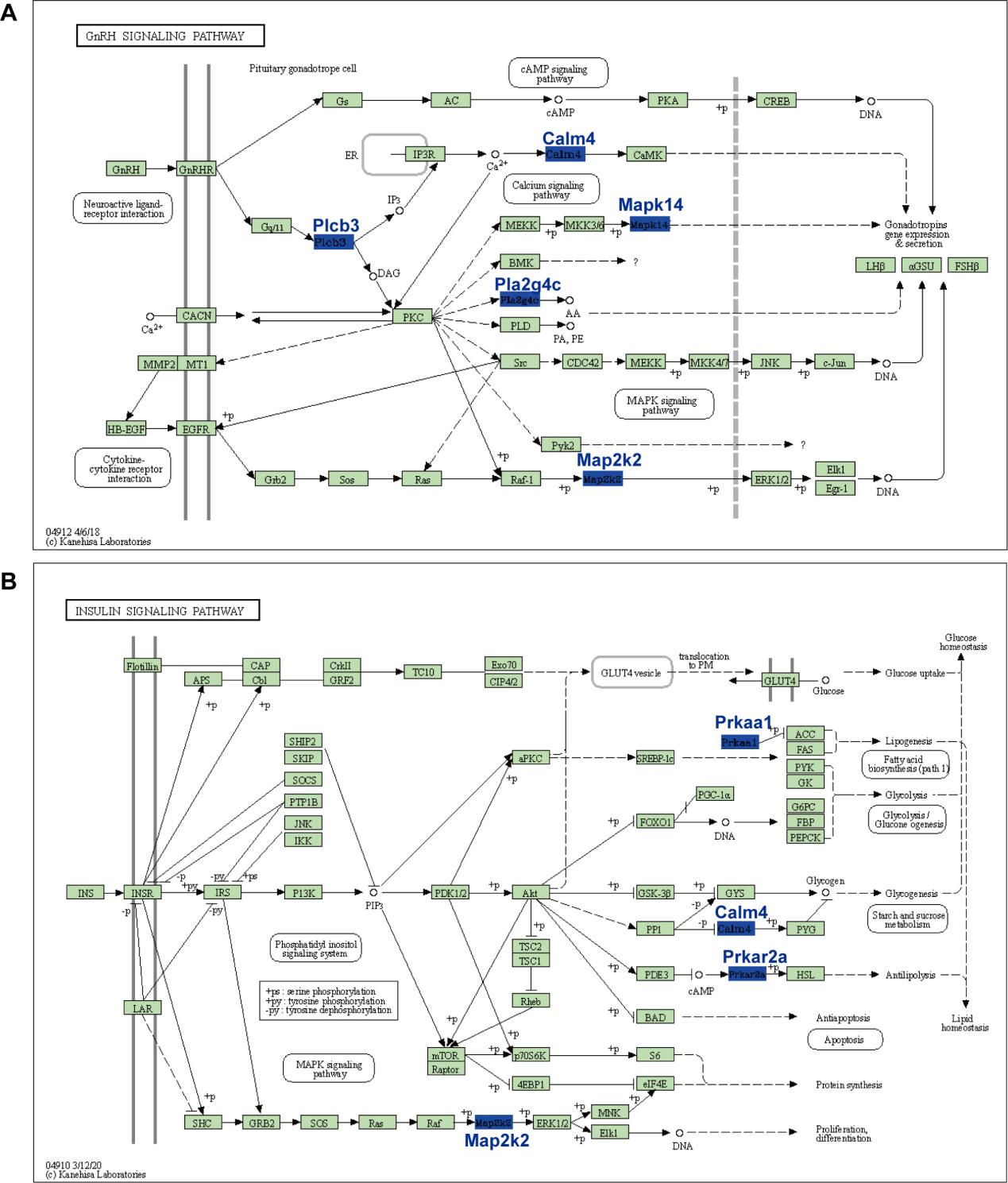


Additional file: **figure 4.** Compared with the CIA group, (A) Mapk14, Plcb3, Map2k2, Pla2g4c and Calm4 in gonadotropin-releasing hormone (GnRH) signaling pathway and (B) targets of Prkar2a, Prkaa1, Map2k2 and Calm4 in insulin signaling pathway are down-regulated in the CIA+Dgal group. The down-regulated DEPs are highlighted in blue and typed out aside.


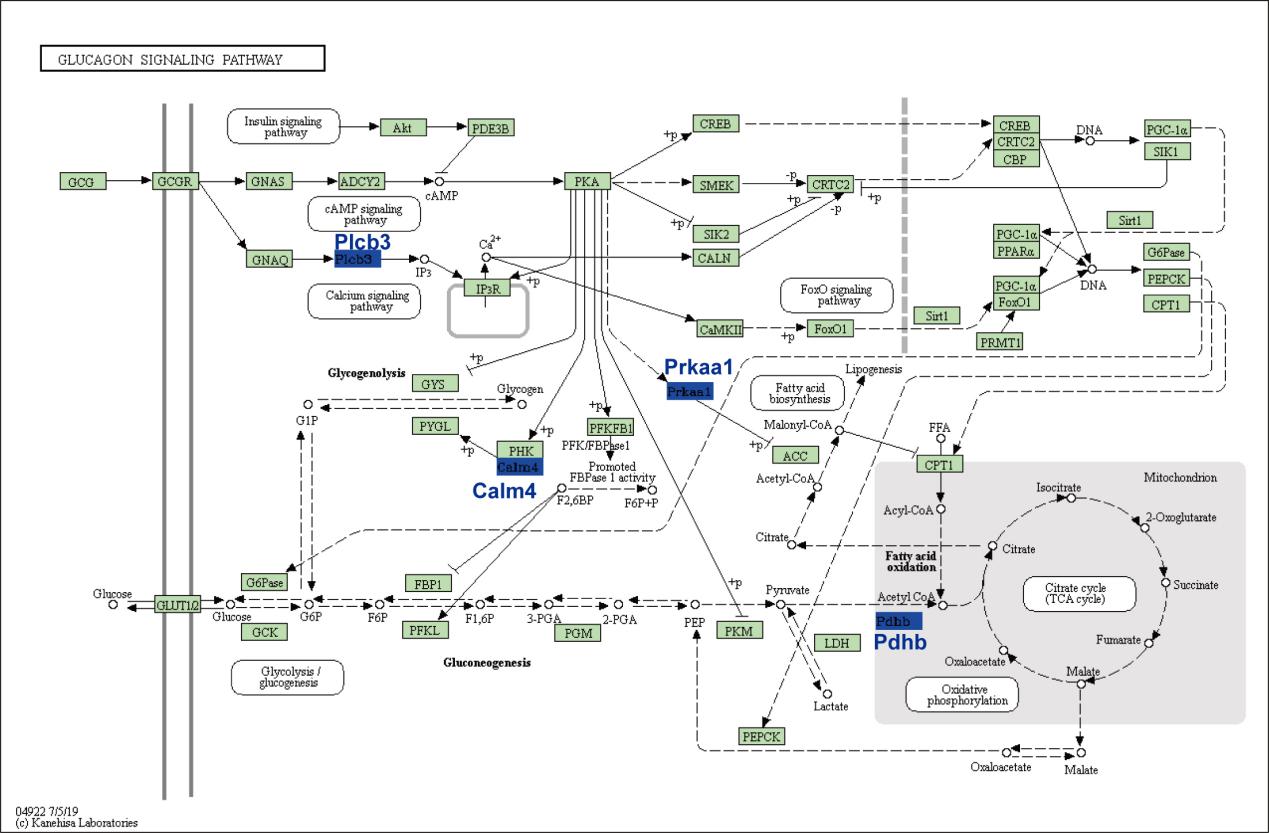


Additional file: **figure 5.** Compared with the CIA group, Plcb3, Prkaa1, Pdhb and Calm4 in Glycogen signaling pathway are down-regulated in the CIA+Dgal group. The down-regulated DEPs are highlighted in blue and typed out aside.


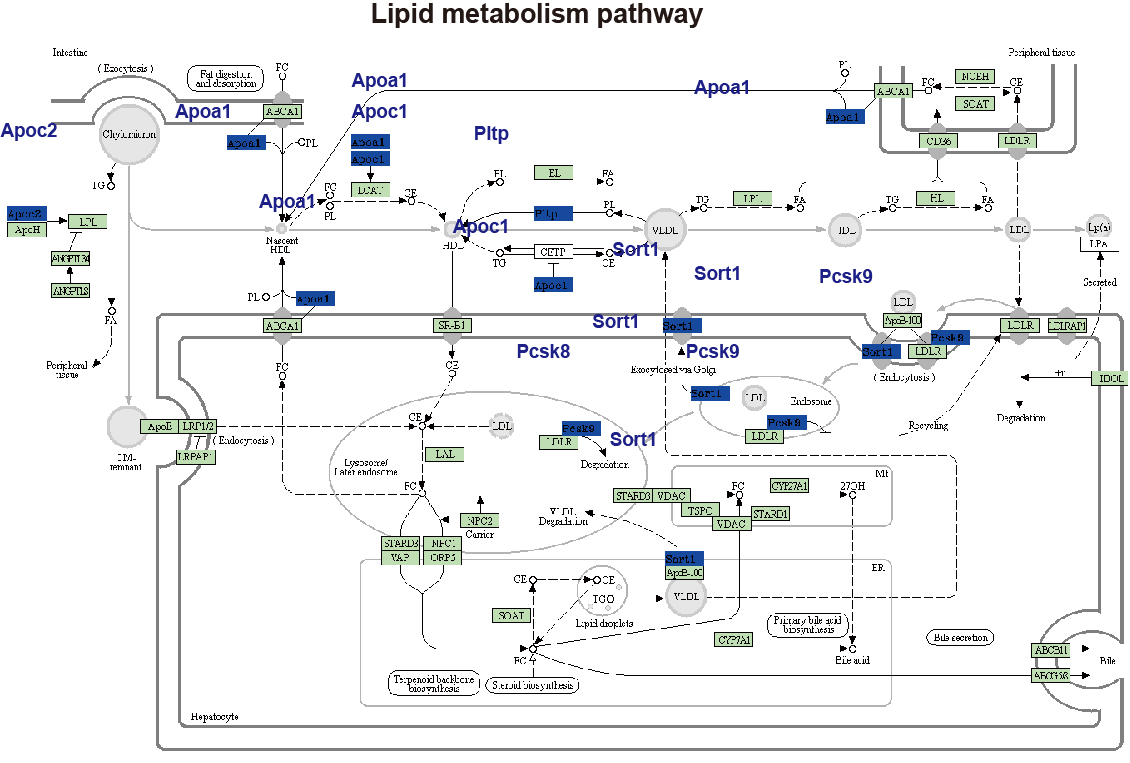


Additional file: **figure 6.** GCNY down-regulated targets of lipid metabolism pathway in CIA+Dgal model. The down-regulated DEPs are highlighted in blue and typed out aside.
